# Prevalence and Molecular Characteristics of Avian Haemosporidian Infection Among Domestic Chickens in Hunan and Guangxi Provinces, China

**DOI:** 10.3390/vetsci13050457

**Published:** 2026-05-08

**Authors:** Haoqing Yang, Jiacheng Tan, Shiquan Lu, Chengjun Xian, Rui Huang, Wei Liu, Dongying Wang

**Affiliations:** 1 College of Animal Science and Technology, Guangxi University, Nanning 530004, China; 13308430348@163.com (H.Y.); 13350750821@163.com (J.T.); m18867029277@163.com (C.X.); ruizhio2002@163.com (R.H.); 2 Guangxi Zhuang Autonomous Region Engineering Research Center of Veterinary Biologics, Nanning 530004, China; 3 College of Veterinary Medicine, Hunan Agricultural University, Changsha 410128, China; 123391@stu.hunau.edu.cn

**Keywords:** *Plasmodium juxtanucleare*, *Leucocytozoon caulleryi*, *Leucocytozoon sabrazesi*, epidemiological characteristics, domestic chicken populations, China

## Abstract

Avian haemosporidian parasites, especially *Plasmodium juxtanucleare*, *Leucocytozoon caulleryi*, and *Leucocytozoon sabrazesi*, represent major threats to poultry health and production. However, there is limited epidemiological information about these pathogens in domestic chickens in Southern China, which hinders effective disease prevention and control. The objective of this study was to conduct a cross-sectional survey to investigate the epidemiological characteristics of these three parasites in Guangxi and Hunan Provinces between June 2024 and December 2025. A total of 941 blood samples were collected from domestic chickens and analyzed using both nested PCR targeting the cytb gene and species-specific PCR assays targeting the *cox*I gene. The overall detection rate of haemosporidian infection was 25.40% (239/941). *P. juxtanucleare* was the most commonly detected species, with a detection rate of 23.59% (222/941), followed by *L. caulleryi* at 1.81% (17/941), while no *L. sabrazesi* infections were identified. Analysis of risk factors showed that chickens older than 90 days had significantly higher detection rates for both *P. juxtanucleare* and *L. caulleryi* compared to younger birds. Additionally, breed-specific differences were noted, with black-bone and partridge chickens showing higher susceptibility to *P. juxtanucleare* than three-yellow chickens. Genetic analysis of *cox*I sequences demonstrated high conservation among *P. juxtanucleare* isolates (99.7–100% similarity) and complete identity among *L. caulleryi* strains. Phylogenetic analysis confirmed that all sequences clustered with the corresponding reference strains from GenBank. This study presents an epidemiological evaluation of these three haemosporidian parasites in domestic chickens from Guangxi and Hunan Provinces, identifying *P. juxtanucleare* as a widespread pathogen and highlighting age and breed as important risk factors. These results emphasize the importance of ongoing monitoring and targeted control measures in the area.

## Simple Summary

This study aimed to examine the epidemiological characteristics of *Plasmodium juxtanucleare*, *Leucocytozoon caulleryi*, and *Leucocytozoon sabrazesi* in domestic chickens from Southern China. A total of 941 blood samples were collected from Hunan and Guangxi Provinces to detect the presence of these parasites using nested PCR and specific PCR methods. The results showed that 23.59% of the chickens were infected with *P. juxtanucleare*, while only 1.81% of chickens were infected with *L. caulleryi,* and no cases of *L. sabrazesi* were detected. Older chickens (>90 days) and certain breeds (black-bone and partridge chickens) exhibited significantly higher infection rates. Genetic analysis revealed that these parasitic strains were highly conserved. This study provides regional epidemiological data and reveals associations with specific ages and breeds.

## 1. Introduction

Avian haemosporidian parasites (Apicomplexa: Haemosporida) are unicellular eukaryotic organisms capable of infecting vertebrate hosts [1]. This diverse group of vector-borne blood parasites predominantly comprises three genera: *Plasmodium*, *Leucocytozoon*, and *Haemoproteus* [2]. Notably, avian haemosporidian parasites have a broad host range, infecting numerous bird species worldwide, from wild passerines to commercially raised poultry [3,4]. These infections can manifest in a spectrum of clinical outcomes, ranging from sub-clinical presentations to severe anemia, weight loss, organ failure, and mortality [5]. Such impacts result in substantial economic losses within the poultry industry and raise conservation concerns for vulnerable avian populations [6]. The biological and pathological attributes of these genera exhibit considerable divergence, of which parasites belonging to *Plasmodium* undergo schizogony within erythrocytes and various endothelial cells, and their transmission predominantly occurs via culicine and anopheline mosquitoes. These parasites are frequently regarded as the most pathogenic among avian heamosporidians, with the capacity to induce fatal infections, particularly in hosts that are not adapted to them [7]. However, species within the genus *Leucocytozoon* undergo exo-erythrocytic schizogony within the parenchymal cells of organs such as the liver, heart, and spleen, and are transmitted by simulid blackflies [8]. Infections caused by species including *Leucocytozoon caulleryi* (*L. caulleryi*) and *Leucocytozoon sabrazesi* (*L. sabrazesi*) can be acute and fatal in chickens, characterized by extensive tissue damage, hemorrhage, and enlargement and discoloration of affected organs [9].

Haemosporidian infections in chickens have been historically documented in China, with *L. caulleryi* and *L. sabrazesi* identified as the predominant species in certain regions [10,11]. Furthermore, the epidemiological profile of *Plasmodium juxtanucleare* (*P. juxtanucleare*), a species known to infect chickens in other regions of Asia and Africa, remains inadequately characterized within China, with only a limited number of molecular studies available [12,13,14]. In recent years, molecular techniques, particularly polymerase chain reaction (PCR) assays targeting mitochondrial genes such as cytochrome b (cytb) and cytochrome c oxidase subunit I (coxI), have significantly advanced the detection and identification of haemosporidian parasites [15,16]. Moreover, these genes have been extensively employed in studies of genetic variation and phylogenetic analysis due to their relatively high rates of sequence variation [17,18].

Various breeds of domestic chickens are reared in Southern China, while the epidemiological data concerning *L. caulleryi*, *L. sabrazesi*, and *P. juxtanucleare* in these areas are still insufficient. Therefore, the aim of this study was to investigate the epidemiological characteristics of *L. caulleryi*, *L. sabrazesi,* and *P. juxtanucleare* in domestic chickens from Guangxi and Hunan Provinces, which will enhance our understanding of the potential risks posed by these parasites to domestic poultry within the studied regions of China.

## 2. Materials and Methods

### 2.1. Sample Collection

**Figure 1:**
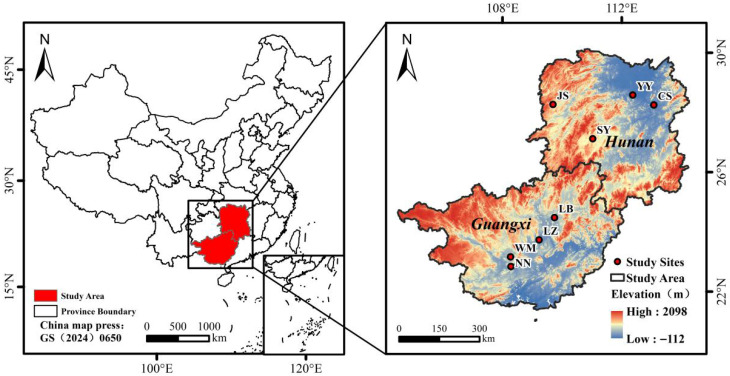
Geographic distribution of sampling sites within Hunan and Guangxi Provinces. The eight sampling locations are denoted by red dots and include CS (Changsha), YY (Yiyang), JS (Jishou), SY (Shaoyang), NN (Nanning), WM (Wuming), LB (Laibin), LZ (Liuzhou). The underlying map was produced using publicly available geographic data.

Between June and December 2024, a total of 941 blood samples were randomly obtained from domestic chickens across eight prefecture-level areas: four located in the Guangxi Zhuang Autonomous Region (Nanning, Wuming, Laibin, and Liuzhou) and four within Hunan Province (Changsha, Yiyang, Jishou, and Shaoyang) (Figure 1). The selected sites represented diverse ecological environments and poultry production systems, encompassing intensive indoor caging, semi-intensive free-range operations, and traditional backyard scavenging practices.

Individual birds were sampled using a haphazard sampling method; approximately 200 μL of blood was aseptically drawn from the wing vein of each chicken using a sterile syringe and immediately transferred into EDTA-2K anticoagulant tubes. Data on host age, breed, and geographic origin were recorded at the time of sampling. For analytical purposes, chickens were categorized into two age groups: less than 90 days and greater than 90 days. These samples included three breeds: black-bone chickens (*n* = 538), three-yellow chickens (*n* = 257), and partridge chickens (*n* = 146). Although the birds were raised under varying production systems, including intensive indoor caging, semi-intensive free-range, and traditional backyard scavenging, the subsequent statistical analyses primarily focused on geographic location, breed, and age as the main variables of interest.

### 2.2. DNA Extraction

Genomic DNA was extracted from 20 μL of each blood specimen utilizing the TIANamp Blood DNA Kit (Tiangen Biotech, Beijing, China) according to the manufacturer’s instructions. The concentration and purity of the extracted DNA were assessed using a NanoDrop spectrophotometer(Thermo Fisher Scientific, Waltham, MA, USA). Subsequently, DNA samples were preserved at −20 °C until their application in PCR.

### 2.3. PCR Amplification and Sequencing

#### 2.3.1. Molecular Detection of Haemosporidian Parasite Infections

An initial screening was performed employing a nested PCR approach targeting an approximately 480 bp fragment of the mitochondrial cytb gene, following the methodology outlined by Iezhova et al. [19]. All primers specifically targeting these genes are shown in Table 1. In each amplification run of the nested PCR, a negative control consisting of nuclease-free water in place of the DNA template was incorporated to detect any possible contamination. PCR was carried out in a total volume of 20 μL, comprising 10 μL of 2× Rapid Taq Master Mix (Vazyme Biotech, Nanjing, China), 0.5 μL of each primer (10 pmol), and 2 μL of template DNA (for the primary PCR) or 1 μL of the primary PCR product (for the nested PCR). The thermal cycling protocol consisted of an initial denaturation step at 94 °C for 3 min, followed by 30 cycles for the primary PCR or 35 cycles for the nested PCR, each cycle including denaturation at 94 °C for 15 s, annealing at 50 °C for 15 s, and extension at 72 °C for 15 s, concluding with a final extension at 72 °C for 5 min.

#### 2.3.2. Species-Specific PCR for *L. caulleryi*, *L. sabrazesi*, and *P. juxtanucleare* Detection

Next, conventional PCR assays were developed to specifically examine the epidemiological characteristics of *L. caulleryi*, *L. sabrazesi*, and *P. juxtanucleare* infections within the collected chicken samples. These assays targeted a partial segment of the coxI gene, employing three distinct primer pairs to detect these three parasites, respectively. Specific primers were designed using SnapGene software (Version 8.2.0), based on the complete mitochondrial genome sequences of *P. juxtanucleare* (GenBank Accession No. NC_008279.1), *L. sabrazesi* (NC_009336.1) and *L. caulleryi* (NC_015304.1) obtained from the GenBank database; basic information on these primers is shown in Table 1. PCRs were performed in a volume of 25 μL, which comprised 12.5 μL of 2× Rapid Taq Master Mix, 0.5 μL of each primer (10 pmol), and 2 μL of template DNA. The thermal cycling conditions included initial denaturation at 94 °C for 4 min, followed by 30 cycles of denaturation at 94 °C for 15 s, annealing at 52 °C for 15 s, and extension at 72 °C for 15 s per kbp, with a final extension step at 72 °C for 5 min. Each species-specific PCR assay included a negative control consisting of nuclease-free water to eliminate the potential for false-positive outcomes. All PCR products were subjected to electrophoresis on 1.5% agarose gels stained with GoodView™ nucleic acid stain. Visualization was performed under ultraviolet illumination using a gel documentation system (Alpha Imager EP, Kodak, Rochester, NY, USA). Positive amplifications exhibiting the anticipated size were subsequently purified and sent to BGI Genomics (Shenzhen, China) for bidirectional Sanger sequencing.

### 2.4. Bioinformatics Analysis

The obtained *cox*I sequences, along with their corresponding reference sequences, were aligned utilizing BioEdit software version 7.2. Subsequent genetic analyses were conducted using DNAStar software version 7.10 (Lasergene DNAStar software). Phylogenetic trees were generated utilizing both Neighbor-Joining (NJ) and Maximum Likelihood (ML) methods in MEGA X version 11 software. Evolutionary distances were calculated according to the Kimura 2-parameter model. The reliability of the constructed phylogenetic trees was evaluated through bootstrap analysis with 1000 replicates.

### 2.5. Statistical Analysis

The collected samples were categorized according to region, age group (<90 days and >90 days), and breed to assess potential infection risks. Infection prevalence, along with 95% confidence intervals (CI), was determined for each location as well as for the overall sample. Chi-square tests in SPSS Statistics version 26.0 software were conducted to compare infection rates across different regions and between the two provinces. Statistical significance was defined as a *p*-value less than 0.05.

## 3. Results

### 3.1. Detection Rates of P. juxtanucleare, L. caulleryi and L. sabrazesi Infections

In the current study, a nested PCR technique was initially utilized to assess the prevalence of haemosporidian infections in domestic chickens from both Guangxi and Hunan Provinces. Out of 941 blood samples collected, 239 samples tested positive for haemosporidian infection, yielding an overall infection rate of 25.40% (239/941, 95% CI: 23.21–29.12). Further PCR assays were conducted to ascertain the detection rate of *P. juxtanucleare*, *L. sabrazesi*, and *L. caulleryi* infections among the haemosporidian-positive samples. As detailed in Table 2, blood samples from 222 (23.59%) and 1.81% (17/941) of the 941 domestic chickens tested positive for *P. juxtanucleare* and *L. caulleryi* infection, respectively. Importantly, none of the clinical samples tested positive for *L. sabrazesi* infection, and no cases of co-infection involving both *P. juxtanucleare* and *L. caulleryi* were detected in any of the clinical specimens.

### 3.2. Risk Factor Analyses

Subsequently, we examined the potential risk factors associated with the detection rate of *P. juxtanucleare* and *L. caulleryi* infections in domestic chickens. As presented in Table 2, the positive rates of *L. caulleryi* among domestic chickens exhibited regional variation, ranging from 0.0% to 7.14%. Notably, the detection rates in Changsha and Jishou cities were significantly higher compared to those samples observed in Nanning city (*p* < 0.05). Moreover, the average detection rate of this parasite in chickens older than 90 days was significantly higher than that observed in chickens younger than 90 days (*p* < 0.05), with respective positive rates of 2.07% (16/773) and 0.60% (1/168). Regarding chicken breeds, the prevalence was 3.16% (17/538) in black-bone chickens, while no positive cases were detected in either three-yellow or partridge chickens. Considering the detection rates of *P. juxtanucleare* across different regions (Table 3), the risks of domestic chickens being infected with this parasite in Nanning, Laibing, Liuzhou, and Changsha were found to be more than twice as high as that in Jishou City (*p* < 0.05). Moreover, the overall risk for chickens older than 90 days was nearly fourfold higher than for those younger than 90 days (OR = 3.73, *p* < 0.01). In light of the positive rates of *P. juxtanucleare* among different chicken breeds, statistical analysis demonstrated that both black-bone chickens and partridge chickens exhibited significantly higher detection rates compared to three-yellow chickens (*p* < 0.05).

### 3.3. Genetic Characteristics and Phylogenetic Analysis of P. juxtanucleare Strains

**Figure 2:**
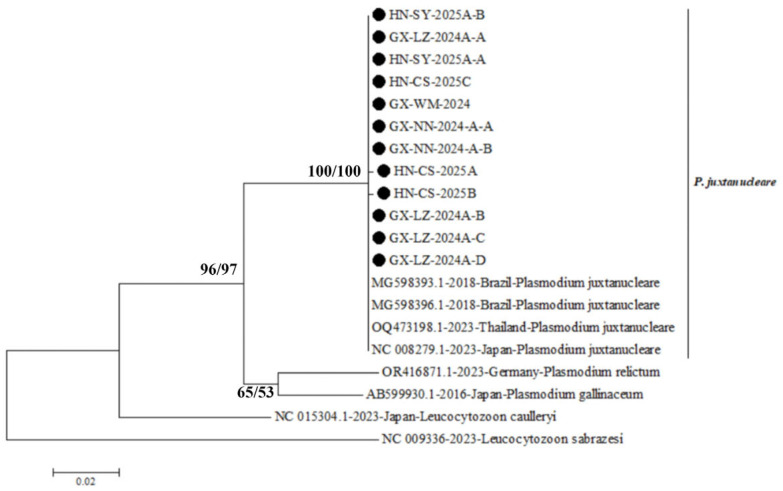
Phylogenetic tree based on the coxI gene sequences was constructed using the NJ and ML methods in MEGA X software, employing *Leucocytozoon sabrazesi* (GenBank accession number NC009336) as the outgroup.

The genetic characteristics of *P. juxtanucleare* strains obtained in this study were subsequently examined. The *cox*I gene sequences from 12 strains were successfully obtained, each comprising an identical fragment of 682 bp in length. Importantly, only two nucleotide substitution sites were identified within the *cox*I gene sequences, specifically at position 515 (T to C) in strain HN-CS-2025A and at position 162 (T to C) in strain HN-CS-2025B. These isolates exhibited a nucleotide sequence similarity ranging from 99.7% to 100.0%, and exhibited a 99.9% to 100.0% similarity when compared to the reference *P. juxtanucleare* strains retrieved from the GenBank database. However, they shared a lower sequence identity (<93.1%) compared to other parasitic strains. Furthermore, a phylogenetic tree was constructed using both the NJ and ML methods in MEGA X software, based on the *cox*I gene sequences. As shown in Figure 2, the findings derived from the ML and NJ phylogenetic trees exhibit a fundamental concordance. All isolates were grouped within a single clade alongside the *P. juxtanucleare* reference strains retrieved from the GenBank database. This clade was distinctly separated from the branches representing other parasites, including *Plasmodium relictum* and *Plasmodium gallinaceum*.

### 3.4. Genetic Features and Phylogenetic Assessment of L. caulleryi Strains

**Figure 3:**
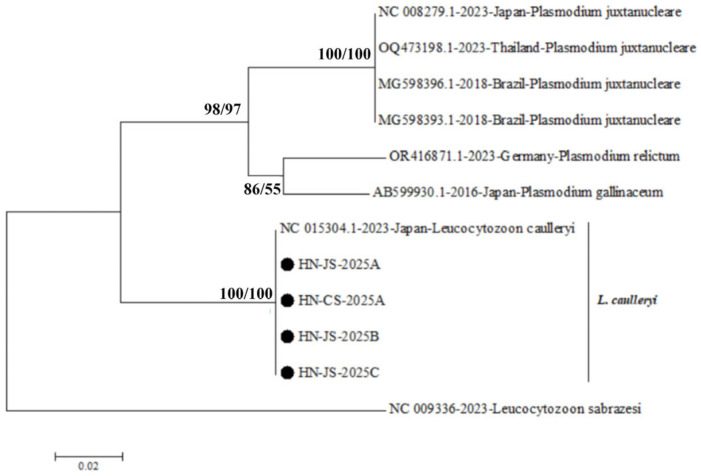
Phylogenetic tree based on the coxI gene sequences was constructed using the NJ and ML methods in MEGA X software, employing *Leucocytozoon sabrazesi* (GenBank accession number NC009336) as the outgroup.

In this study, the *cox*I gene sequences of only four strains of *L. caulleryi* were successfully sequenced, each measuring 754 bp in length. Notably, these four strains showed a complete match in (100%) nucleotide sequence identity with each other, and had the greatest similarity (99.7%) to the reference *L. caulleryi* strain (GenBank accession number: NC015304), while they exhibited a less than 90% sequence similarity with other parasites. Similarly, the four strains obtained in this study were clustered into a single clade with the reference *L. caulleryi* strain, distinctly separated from the branches of other parasites (Figure 3).

## 4. Discussion

Avian haemosporidians are frequently present in poultry populations around the world. In particular, the widespread occurrence of certain species, such as *P. juxtanucleare* and *L. caulleryi,* presents a major risk to the development of the chicken industry. However, the prevalence of these parasites is frequently overlooked due to the scarcity of epidemiological research conducted in China [10,12]. Investigation of the prevalence of the aforementioned pathogens is essential to prevent and manage these diseases. Therefore, this study aimed to comprehensively examine the epidemiological characteristics of three haemosporidian species (*P. juxtanucleare, L. sabrazesi,* and *L. caulleryi*) in domestic chickens from Southern China. The nested-PCR analysis showed that the overall detection rate of haemosporidian infection among the collected samples was 25.40% (239/941, 95% CI: 23.2–29.1). Notably, the detection rate observed in this study was lower than that reported in Hainan Province in 2017 (77.87%, 95/122) [20] and in Beijing city between 2014 and 2015 (88.7%, 165/186) [21]. This discrepancy could be attributed to differences in the time of investigation, sample size, growth stages, and other factors.

Additionally, the detection rates for *P. juxtanucleare, L. caulleryi,* and *L. sabrazesi* were 23.59% (222/941), 1.81% (17/941), and 0%, respectively. Similarly, Xuan et al. reported that the detection rate of *P. juxtanucleare* infection was significantly higher compared to the other three haemosporidian parasites among chickens in Thailand [22]. Two factors may explain this observation: (1) The primary insect vectors responsible for transmitting *P. juxtanucleare, L. sabrazesi,* and *L. caulleryi* wereCulex saltanensis, Culicoides, and Culicoides arakawae, respectively [13,23]. It is hypothesized that the greater prevalence of Culex saltanensis in the examined areas contributes to an increased infection rate of *P. juxtanucleare*. (2) *P. juxtanucleare* is capable of infecting various bird species, including chickens and other birds [6], which may help in the transmission of this parasite. Moreover, chickens older than 90 days had a higher prevalence of *P. juxtanucleare* infection compared to those younger than 90 days, indicating that age is a risk factor for a high prevalence of *P. juxtanucleare*. Notably, similar patterns have been observed with other parasites in bird species, such as *Toxoplasma gondii* in both chickens and ducks [24,25].

This study also showed that black-bone chickens and partridge chickens had significantly higher detection rates of *P. juxtanucleare* infection than three-yellow chickens, with black-bone chickens having the highest prevalence of *L. caulleryi* infection among the three types of chickens. Several factors may contribute to this phenomenon: (1) Black-bone chickens and partridge chickens are typically raised in free-range or semi-free-range systems, which can increase their opportunities of coming into contact with vector organisms like blood-sucking insects. (2) Black-boned chickens and partridge chickens may be more likely to act as recessive carriers, harboring insects for extended periods without displaying any clinical symptoms.

In recent years, there has been significant interest in the epidemiological features of Haemosporida [26,27], but relevant data remains scarce in China [10,12]. In the present study, the coxI gene sequences of twelve *P. juxtanucleare*- and four *L. caulleryi*-positive samples were successfully sequenced to analyze their genetic variation. Notably, the nucleotide variations in the coxI sequences were between 0 and 0.3% for *P. juxtanucleare* and 0% for *L. sabrazesi strains*. Similarly, Dhaayanti et al. reported that nine *P. juxtanucleare* strains obtained from Indonesia exhibited a genetic distance of 0 to 1% based on the cytb gene [15]. These findings highlight the highly conserved nature of *P. juxtanucleare* strains. However, owing to the small number of *L. caulleryi* strains (*n* = 4) obtained in this study, additional research will be conducted to gather more samples and examine their genetic traits. Additional phylogenetic analysis showed that all *P. juxtanucleare* and *L. caulleryi* strains identified in this study, along with their respective reference strains collected from the GenBank database, clustered within a single clade and were randomly dispersed within it.

It should be noted that there are certain limitations in this study. Firstly, the limited quantity of sampling points and samples may not accurately represent the true prevalence of the three pathogens in the regions of Guangxi and Hunan, China. Secondly, this study employed specific PCR techniques to detect the presence of *P. juxtanucleare*, *L. caulleryi*, and *L. sabrazesi*, respectively. However, conventional PCR demonstrated lower sensitivity relative to the real-time PCR method, potentially leading to an underestimation of the true positive rate and the prevalence of mixed infections. Thirdly, insect vectors play a crucial role in the transmission of *P. juxtanucleare*, *L. sabrazesi*, and *L. caulleryi* among poultry populations [13,23], and this study did not investigate the prevalence of these three parasites in insect vectors. Fourthly, due to the limited sample size of *L. caulleryi* (*n* = 4) in this study, the findings may not comprehensively represent the actual genetic diversity of this parasite within the examined regions.

## 5. Conclusions

This study constitutes an inaugural comprehensive epidemiological analysis of *P. juxtanucleare*, *L. caulleryi*, and *L. sabrazesi* infections in domestic chickens within the provinces of Guangxi and Hunan in Southern China. The results show that *P. juxtanucleare* is commonly found (23.59%) in these areas, while *L. caulleryi* is much less common (1.81%), and *L. sabrazesi* was not found at all. Age and breed were significant risk factors, with older chickens and certain breeds (black-bone and partridge chickens) being more vulnerable. Genetic analysis revealed that the strains of both *P. juxtanucleare* and *L. caulleryi* are highly conserved based on genetic analysis of the coxI gene. These results underscore the critical need for continuous surveillance of haemosporidian infections within poultry farming operations in Southern China.

## Figures and Tables

**Table 1:** Primers used for PCR amplification in this study.

Primer	Sequence 5′-3′	Length	Purpose	Reference Sequence
HAEMN-F	CATATATTAAGAGAATTATGGAG	581 bp	Primary PCR for detecting Haemosporidian	LecaoM_p03
HAEMN-R	AGAGGTGTAGCATATCTATCTAC			
HAEM-F	ATGGTGCTTTCGATATATGCATG	525 bp	Second PCR for detecting Haemosporidian	LecaoM_p03
HAEM-R	GCATTATCTGGATGTGATAATGGT			
PF11	CCAAGGAAATGCATAGGTAA	782 bp	Detection of *P. juxtanucleare*	NC_008279.1
PR11	GCAAAAGGATTAACACTTGG			
CLcox12-F	GCCTGGATTATTTGGTGGTTT	1131 bp	Detection of *Leucocytozoon caulleryi*	NC_015304.1
CLcox12-R	GCGTCTGGATAATCGGGAAT			
LScoxI-F	GATCTTCTTCAATGTAATGCCTGGA	1193 bp	Detection of *Leucocytozoon sabrazesi*	NC_009336.1
LScoxI-R	TGGTAGTTGATCCAAGAGAACATAC			

**Table 2:** The detection rates of *L. caulleryi* infection among domestic chickens.

Factor	Category	No. Tested	No. Positive	Prevalence (%) (95% CI)	*p*-Value	OR (95%CI)
Region	Changsha	148	4	2.70 (0.08–5.30)	0.961	2.69 (0.30–24.47)
	Yiyang	64	0	-		
	Jishou	168	12	7.14 (3.48–11.43)	0.043	7.46 (0.96–58.37)
	Shaoyang	184	0	-		
	Nanning	98	1	1.02 (0.52–3.03)		Reference
	Wuming	72	0	-		
	Laibing	80	0	-		
	Liuzhou	127	0	-		
Age	<90 days	168	1	0.60 (0.13–1.62)		Reference
	>90 days	773	16	2.07 (1.07–3.06)	0.224	3.53 (0.47–28.62)
Breed	Black-bone chicken	538	17	3.16 (1.68–4.64)		
	Three-yellow chicken	257	0	-		
	Partridge chicken	146	0	-		
	In total	941	17	1.81 (0.9–2.61)		

**Table 3:** The detection rates of *P. juxtanucleare* infection among domestic chickens.

Factor	Category	No. Tested	No. Positive	Prevalence (%) (95% CI)	*p*-Value	OR (95%CI)
Region	Changsha	148	94	63.51 (55.75–71.26)	<0.01	17.76 (9.48–33.26)
	Yiyang	64	0	-		
	Jishou	168	15	8.93 (4.62–13.24)		Reference
	Shaoyang	184	21	11.41 (6.82–16.00)	0.443	1.31 (0.65–2.64)
	Nanning	98	25	25.51 (16.88–34.13)	<0.01	3.49 (1.74–7.02)
	Wuming	72	11	15.28 (6.96–23.59)	0.152	1.84 (0.80–4.23)
	Laibing	80	15	18.75 (10.18–27.30)	0.0291	2.35 (1.09–5.10)
	Liuzhou	127	41	32.28 (24.15–40.41)	<0.01	4.85 (2.55–9.29)
Age	<90 days	168	15	8.93 (4.62–13.24)		Reference
	>90 days	773	207	26.78 (23.66–29.90)	<0.01	3.73 (2.14–6.49)
Breed	Black-bone chicken	538	156	29.00 (25.17–32.83)	<0.01	2.68 (1.78–4.02)
	Three-yellow chicken	257	34	13.23 (9.09–17.32)		Reference
	Partridge chicken	146	32	21.92 (15.21–28.63)	0.0248	1.84 (1.08–3.14)
	In total	941	222	23.59 (20.88–26.30)		

## Data Availability

The data presented in this study are openly available in NCBI at https://www.ncbi.nlm.nih.gov/Genbank/update.html (accessed on 5 May 2026) reference number PZ188674 to PZ188689.
